# Prevalence of preeclampsia and algorithm of adverse foeto-maternal risk factors among pregnant women in the Central Region of Ghana: A multicentre prospective cross-sectional study

**DOI:** 10.1371/journal.pone.0288079

**Published:** 2023-06-29

**Authors:** Enoch Odame Anto, Wina Ivy Ofori Boadu, Ezekiel Ansah, Augustine Tawiah, Joseph Frimpong, Valentine Christian Kodzo Tsatsu Tamakloe, Emmanuel Ekow Korsah, Emmanuel Acheampong, Evans Adu Asamoah, Stephen Opoku, Eric Adua, Ebenezer Afrifa-Yamoah, Max Efui Annani-Akollor, Agartha Odame Anto, Christian Obirikorang

**Affiliations:** 1 Faculty of Allied Health Sciences, Department of Medical Diagnostics, College of Health Sciences, Kwame Nkrumah University of Science and Technology, Kumasi, Ghana; 2 School of Medical and Health Sciences, Edith Cowan University, Joondalup Drive, Perth, Australia; 3 Centre for Precision Health, ECU Strategic Research Centre, Edith Cowan University, Perth, Australia; 4 Department of Obstetrics and Gynaecology, Komfo Anokye Teaching Hospital, Kumasi, Ghana; 5 Department of Molecular Medicine, School of Medicine and Dentistry, College of Health Science, Kwame Nkrumah University of Science and Technology, Kumasi, Ghana; 6 Rural Clinical School, Medicine and Health, University of New South Wales, Sydney, NSW, Australia; 7 School of Science, Edith Cowan University, Joondalup, Australia; 8 Department of Obstetrics and Gynaecology, Ho Teaching Hospital, Ho, Ghana; Arba Minch University, ETHIOPIA

## Abstract

**Background:**

Preeclampsia is a leading cause of foeto-maternal deaths especially in Sub-Saharan Africa. However, the prevalence and risk factors of preeclampsia are scarce in the Central region of Ghana with previous study assessing individual independent risk factors. This study determined the prevalence and algorithm of adverse foeto-maternal risk factors of preeclampsia.

**Methods:**

This multi-centre prospective cross-sectional study was conducted from October 2021 to October 2022 at the Mercy Women’s Catholic Hospital and Fynba Health Centre in Central region, Ghana. A total of 1,259 pregnant women were randomly sampled and their sociodemographic, clinical history, obstetrics and labour outcomes were recorded. Logistic regression analysis using SPSS version 26 was performed to identify risk factors of preeclampsia.

**Results:**

Of the 1,259 pregnant women, 1174 were finally included in the study. The prevalence of preeclampsia was 8.8% (103/1174). Preeclampsia was common among 20–29 years age group, those who had completed basic education, had informal occupation, multigravida and multiparous. Being primigravida [aOR = 1.95, 95% CI (1.03–3.71), *p* = 0.042], having previous history of caesarean section [aOR = 4.48, 95% CI (2.89–6.93), *p*<0.001], foetal growth restriction [aOR = 3.42, 95% CI (1.72–6.77), *p*<0.001] and birth asphyxia [aOR = 27.14, 95% CI (1.80–409.83), *p* = 0.017] were the independent risk factors of preeclampsia. Pregnant women exhibiting a combination of primigravida, previous caesarean section and foetal growth restriction were the highest risk for preeclampsia [aOR = 39.42, 95% CI (8.88–175.07, *p*<0.001] compared to having either two or one of these factors.

**Conclusion:**

Preeclampsia is increasing among pregnant women in the Central region of Ghana. Pregnant women being primigravida with foetal growth restriction and previous history of caesarean section are the highest risk population likely to develop preeclampsia with neonates more likely to suffer adverse birth outcome such as birth asphyxia. Targeted preventive measures of preeclampsia should be created for pregnant women co-existing with multiple risk factors.

## Introduction

Preeclampsia, a hypertensive disorder of pregnancy (HDP) is a major health burden in the obstetric population [1]. Preeclampsia (PE) is a primary cause of maternal and newborn death and morbidity, affecting 2–5% of all pregnancies worldwide [2].

Preeclampsia is characterised by new onset of hypertension with and without proteinuria and multiple organ dysfunction that appears at or after 20 weeks of pregnancy [3]. Despite the understood aetiology, PE condition is linked to the failure of trophoblastic invasion of maternal spiral arteries, which increases uterine artery vascular resistance and decreases uteroplacental blood flow [4,5]. It is seven times more common in underdeveloped countries than in developed countries [6].

Preeclampsia prevalence in developing nations ranges from 1.8% to 16.7% [7]. A Longitudinal prospective analytical survey showed the prevalence of PE in Ghana ranged between 6.55% and 7.03% from 2006 to 2009 [8]. According to data from Ghana in 2014, women with hypertensive disorders of pregnancy had a high prevalence of PE (48.8%) [9]. Thus, the prevalence of preeclampsia is poorly characterized, and data on this condition is limited.

Women in impoverished nations are 33 times more likely to die from maternal-related causes than women in developed nations [10]. In Ghana’s Central Region, lipid abnormalities and maternal obesity are associated with preeclampsia among pregnant women in the Cape Coast Metropolis [11]. The metabolic and lifestyle characteristics such as alcohol consumption and dietary lifestyle such as salted fish consumption may also contribute to this pregnancy complication [12–14]. Several studies have identified advanced maternal age, maternal body mass index (BMI), parity, multiple gestation, history of diabetes mellitus, pregnancy hypertension, and gestational diabetes as clinical risk factors for preeclampsia [15–17] but have reported and validated these as individual risk factors. Despite this, only a few studies have investigated the prevalence of PE in Ghana. Again, no published data is available on the prevalence of preeclampsia in Mankessim, the Central Region of Ghana. Not only have previous studies assessed individual risk factors but also, they have focused less on pregnant women with multiple risk factors of preeclampsia.

We assessed the prevalence of preeclampsia among pregnant women in Mankessim, the Central region of Ghana as well as identified the odds of preeclampsia among pregnant women exhibiting multiple risk factors. This finding will contribute to the development of health policy, including identification and clinical follow-up of women at risk of preeclampsia, and decrease adverse pregnancy outcomes among mothers as well.

## Materials and methods

### Study design/setting

This was a multicentre prospective cross-sectional study conducted at the Mercy Women’s Catholic Hospital (MWCH) and Fynba Health Centre, Ghana from October 2021 to October 2022. The Mercy Women’s Catholic Hospital (MWCH) and Fynba Health Centre are health institutions located in the Mankessim a town in Mfantseman West Constituency in the central region of Ghana. MWCH which has a 133-bed capacity serves as a referral centre for the surrounding towns in the Mfantseman West Constituency whereas Fynba Health Centre is a supporting health centre with a 50-bed capacity.

### Study population and participant selection

**Fig 1:**
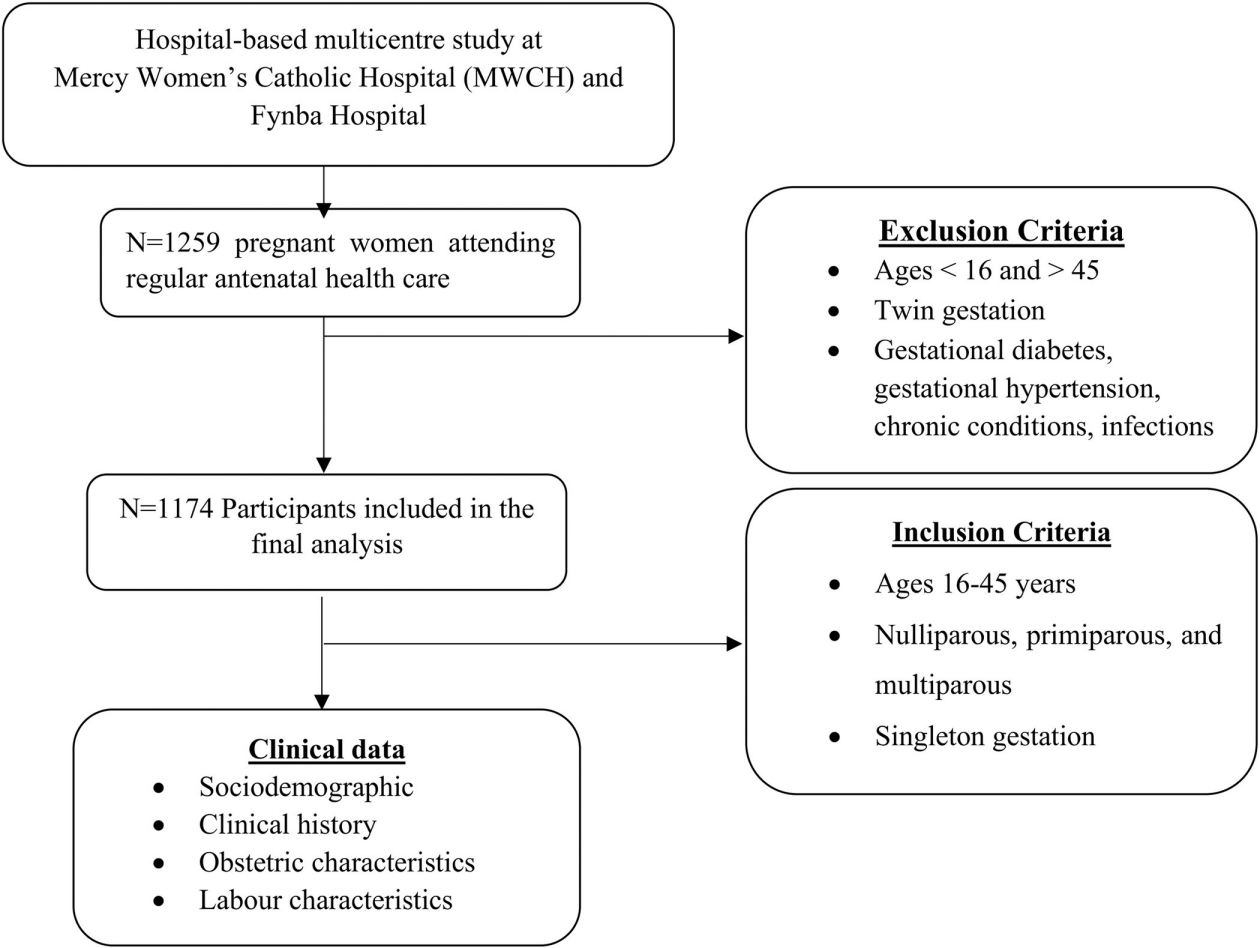
Study participants.

A simple randomised sampling technique was used to recruit 1,259 pregnant women aged 16–45 years who were at or after 20 weeks of gestation and attending regular antepartum care. Of the 1,259 pregnant women’s data obtained, 85 pregnant women were excluded from the study as they did not meet the inclusion criteria and a total of 1,174 participants who were mostly singleton pregnant women were finally included in the study. Women with twin pregnancies, below 16 years of age, advanced maternal age (>45 years), gestational diabetes, gestational hypertension, obesity, smoking, alcoholism, receiving magnesium sulphate (MgSO4) treatment, receiving anti-hypertensive, sexually transmitted infections, sickle cell anaemia and other known chronic condition were excluded from the study. Sociodemographic characteristics (age, level of education, occupation), obstetric characteristics (gravidity, parity, antenatal care visit, gestational age, antepartum haemorrhage, and foetal growth restriction), clinical history (gestational diabetes, gestational hypertension, chronic conditions, infections) and labour characteristics such as (foetal gender, birth weight, foetal body length, foetal head circumference, birth abnormality, foetal lie and foetal presentation, mode of delivery, post-partum haemorrhage and birth asphyxia) were collected from participants using structured closed-ended questionnaires and patients folders and with patients hospital registry **(Fig 1)**.

Preeclampsia was defined based on the revised definition by the International Society for the study of Hypertension in pregnancy (ISSHP) as a new-onset of gestational hypertension (≥140 mmHg systolic/≥90 mmHg diastolic) developed at or after 20 weeks gestation and with new-onset of at least either one of proteinuria (spot check urine protein >30 mg/ mmol [0.3 mg/mg] or >300 mg/day or at least 1 g/L (‘2+’ using dipstick testing) or without proteinuria but the involvements of maternal organ dysfunctions (neurological complications, pulmonary oedema, haematological complications, liver involvement or acute kidney injury) and or uteroplacental dysfunction [18]. Diagnosis of preeclampsia was done by a consultant obstetrician and gynaecologist. Uteroplacental dysfunction was evaluated with ultrasound assessment of foetal growth and umbilical artery Doppler velocimetry or cerebroplacental ratio measurements to assess blood flow redistribution in placental insufficiency according to the International Society for Ultrasound in Obstetrics and Gynaecology guidelines [19].

### Ethical consideration

Approval for this study was given by the Committee on Human Research, Publications and Ethics (CHRPE) (CHRPE/AP/291/22), School of Medicine and Dentistry, Kwame Nkrumah University of Science and Technology (KNUST). Written informed consent in the form of signature and fingerprint was obtained from all participants and Legally Authorised Representatives before the commencement of the study. The consent process involved explaining the study’s objectives, procedures, risks, benefits, and voluntary nature, allowing participants to make an informed decision. Strict measures ensured confidentiality and privacy, with anonymized and securely stored data accessible only to authorized researchers involved in this study. This study was conducted in accordance with the guidelines of the Helsinki Declaration [20].

### Clinical measurements

#### Blood pressure measurement

The systolic blood pressure (SBP) and diastolic blood pressure (DBP) of the participants were measured by trained medical personnel using an automated blood pressure recorder (Omron HEM 7120, Japan), in accordance with recommendations of the American Heart Association [21] while they were seated in a comfortable position after at least 15 min of rest.

#### Urine collection and proteinuria estimation

Participants were asked to provide 10–15 ml of early morning urine in sterile leak proof containers. Due to the difficulty of obtaining a 24-hour urine collection, proteinuria was measured using a urine reagent dipstick (a semi-quantitative colour scale on the URIT 2VPG Medical electronic Co., Ltd. China). These strips categorize proteinuria as negative, trace, 0.3 g/L, 1.0g/L, or 3.0 g/L, corresponding to negative, trace, 1+, 2+, and 3+, respectively; a positive test was considered to be ≥ 0.3 g/L (≥ 1+) [22].

### Definition of clinical terms

Gravidity was defined as the number of pregnancies of a woman regardless of the outcome [23]. Parity was defined as the total number of births after 20 weeks of gestation, including live birth and stillbirth [24]. Foetal growth restriction (FGR) was defined as the failure of a foetus to grow according to its genetically determined growth potential [25]. Birth Asphyxia was defined as the failure to initiate and sustain breathing or spontaneous breathing at birth [26]. Caesarean Section was defined as delivery through an open abdominal incision and an incision in the uterus [27]. Postpartum haemorrhage was defined as > 1000 mL blood loss within 24 hours after childbirth, regardless of the mode of delivery [28]. Antepartum haemorrhage was defined as bleeding from the vagina after 24 weeks [29].

### Statistical analyses

The collected data obtained were entered, coded, edited, and cleaned in Microsoft Excel 2019. All statistical analyses were performed using the Statistical Package for the Social Sciences (SPSS) Version 26.0 (Chicago IL, USA) and GraphPad Prism version 8.0.1 (GraphPad Software, San Diego California USA, www.graphpad.com). Descriptive statistics were used to present the baseline data variables. Categorical data were presented as frequencies and percentages. Chi-square test/Fischer’s Exact test and the univariate followed by multivariate logistic regression analysis were employed to test for associations and the strength thereof between the dependent variable (preeclampsia) and independent variables. The p-values less than 0.05 were considered statistically significant for all analyses.

## Results

**Table 1** shows the sociodemographic and maternal obstetric characteristics of the study participants. Of the 1174 study participants, most were aged 20–29 years 576(49.1%) while only 43(3.7%) were 40 years or older. Although 138(11.8%) of the women had no formal education, majority had received basic education 688(58.6%), 209(17.8%) had secondary education and 139(11.8%) had tertiary education. More than half of the pregnant women were multigravida 625(53.2%) followed by those who were primigravida 298(25.4%) and grand multigravida 251(21.4%). In addition, majority of the pregnant women 794(67.6%) worked in the informal sector compared to 157(13.4%) who were formal sector workers. Most of them were multiparous 523(44.5%). A higher proportion 1066(90.8%) of the women had more than four antenatal care visits. Significant association between education level (p = 0.0290), occupation (p = 0.0070) and preeclampsia were found. However, there were no significant association between participants age groups (p = 0.1980), gravida (p = 0.1170), parity (p = 0.3580), ANC visit (p = 0.8600) and preeclampsia (Table 1).

**Table 1:** Sociodemographic and maternal obstetric characteristics of study participants.

Variable	Total (n = 1174)	NTN-PW (n = 1071)	PE (n = 103)	p- value
**Age Group (years)**				0.1980
< 20	126(10.7)	117(10.9)	9(8.7)	
20–29	576(49.1)	531(49.6)	45(43.7)	
30–39	429(36.5)	387(36.1)	42(40.8)	
≥ 40	43(3.7)	36(3.4)	7(6.8)	
**Level of education**				**0.0290**
No education	138(11.8)	124(11.6)	14(13.6)	
Basic	688(58.6)	640(59.5)	48(46.6)	
Secondary	209(17.8)	188(17.5)	21(20.4)	
Tertiary	139(11.8)	119(11.1)	20(19.4)	
**Occupation**				**0.0070**
Formal	157(13.4)	134(12.5)	23(22.3)	
Informal	794(67.6)	726(67.8)	68(66.0)	
Unemployed	223(19.0)	211(19.7)	12(11.7)	
**Gravidity**				0.1170
Primigravida	298(25.4)	264(24.6)	34(33.0)	
Multigravida	625(53.2)	579(54.1)	46(44.7)	
Grand multigravida	251(21.4)	228(21.3)	23(22.3)	
**Parity**				0.3580
Nulliparous	363(30.9)	325(30.3)	38(36.9)	
Primiparous	288(24.6)	266(24.8)	23(22.3)	
Multiparous	523(44.5)	480(44.8)	42(40.8)	
**ANC visit**				0.8600
< 4	108(9.2)	98(9.2)	10(9.7)	
≥ 4	1066(90.8)	973(90.8)	93(90.3)	

Data is presented as frequency (%); Chi square/ Fisher’s exact test, p-value < 0.05 was considered statistically significant for preeclampsia and normotensive mothers. NTN, Normotensive; PW, Pregnant Women; PE, Preeclampsia; ANC, Antenatal Care.

**Fig 2:**
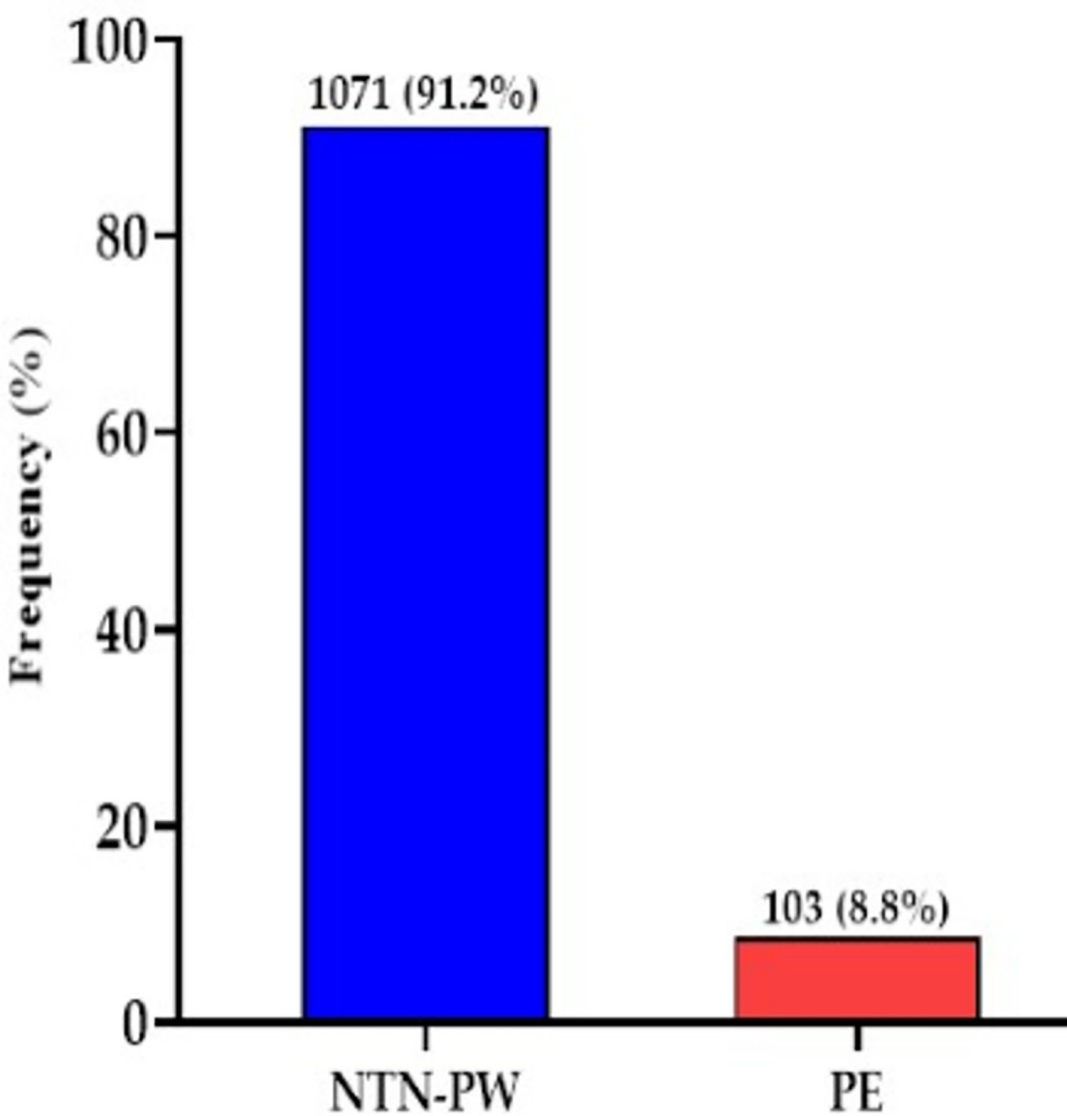
Prevalence of preeclampsia among the study participants.

The prevalence of preeclampsia among the study participants was 8.8% (103/1174) whereas91.2% (1071/1174) were normotensive pregnant women **(Fig 2).**

**Table 2** shows association of adverse foeto-maternal complications with preeclampsia among the study participants. This study found a significant association between birth asphyxia (*p* = 0.0410), mode of delivery (*p*<0.0001) and FGR (*p*<0.0001) with preeclampsia. On the contrary, there were no significant association between prolonged labour (*p* = 0.8120), postpartum haemorrhage (PPH) (*p* = 0.9990) and antepartum haemorrhage (APH) (*p* = 0.0900), and preeclampsia **(Table 2).**

**Table 2:** Adverse foeto-maternal complications with preeclampsia among the study participants.

Variable	Total (n = 1174)	NTN-PW (n = 1071)	PE (n = 103)	*p*- value
**Birth asphyxia**				**0.0410**
No	1168(99.5)	1067(99.6)	101(98.1)	
Yes	6(0.5)	4(0.4)	2(1.9)	
**Prolonged labour**				0.8120
No	1118(95.2)	1019(95.1)	99(96.1)	
Yes	56(4.8)	52(4.8)	4(3.9)	
**PPH**				0.9990
No	1094(93.2)	998(93.2)	24(23.3)	
Yes	80(6.8)	73(6.8)	79(76.7)	
**APH**				0.0900
No	1168(99.5)	1067(99.6)	101(98.1)	
Yes	6(0.5)	4(0.4)	2(1.9)	
**Mode of delivery**				**<0.0001**
Previous SVD	798(68.0)	762(71.1)	36(35.0)	
Previous C/S	376(32.0)	309(28.9)	67(65.0)	
**FGR**				**<0.0001**
Yes	101(8.6)	80(7.5)	21(20.4)	
No	1073(91.4)	991(92.5)	82(79.6)	

Data is presented as frequency (%); Chi square/ Fisher’s exact test, p-value < 0.05 was considered statistically significant for preeclampsia and normotensive mothers. NTN, Normotensive; PW, Pregnant Women; PE, Preeclampsia; PPH, Postpartum Haemorrhage; APH, Antepartum Haemorrhage; SVD, Spontaneous Vaginal Delivery; C/S, Caesarean section; FGR, Foetal growth restriction.

**Table 3** depicts sociodemographic and maternal obstetric predictors of preeclampsia among study participants. In a univariate logistic regression model, basic education, informal occupation, unemployed and primigravida were predictors of preeclampsia. In multivariate logistic regression model, primigravida [Adjusted odds ratio (aOR) = 1.95, 95% CI (1.03–3.71), *p* = 0.0420] remained significant and was the independent predictor of preeclampsia **(Table 3).**

**Table 3:** Logistic regression model of sociodemographic and maternal obstetric predictors of preeclampsia among study participants.

Variable	cOR (95% CI)	p-value	aOR (95% CI)	p-value
**Age group (years)**				
20–29	1.00		-	-
< 20	0.91(0.43–1.91)	0.7980	-	-
30–39	1.28(0.82–1.99)	0.2710	-	-
≥ 40	2.29(0.97–5.45)	0.0600	-	-
**Level of education**				
Tertiary	1.00		1.00	
No education	0.48(0.20–1.14)	0.0960	0.76(0.20–2.86)	0.6840
Basic	0.45(0.256–0.779)	**0.0050**	0.89(0.29–2.71)	0.8410
Secondary	0.67(0.35–1.28)	0.2210	1.21(0.43–3.40)	0.7250
**Occupation**				
Formal	1.00		1.00	
Informal	0.55(0.33–0.91)	**0.0200**	0.79(0.29–2.14)	0.6380
Unemployed	0.33(0.16–0.69)	**0.0030**	0.49(0.15–1.61)	0.2370
**Gravidity**				
Multigravida	1.00		1.00	
Primigravida	1.62(1.02–2.59)	**0.0420**	1.82(1.01–3.29)	**0.0420**
Grand multigravida	1.26(0.75–2.13)	0.3800	0.88(0.44–1.74)	0.7070
**Parity**				
Primiparous	1.00		-	-
Nulliparous	1.41(0.82–2.45)	0.2170	-	-
Multiparous	1.06(0.62–1.81)	0.8370	-	-
**ANC Visit (per number of times)**				
≥4	1.00		-	-
<4	1.04(0.52–2.06)	0.9180	-	-

PE, Preeclampsia; cOR, Crude Odd ratio; CI, Confidence interval; aOR, Adjusted Odd ratio; inf, infinity; 1.00, ref. Binary logistic regression analysis performed to obtain odd ratios. *p*-value of < 0.05 was considered statistically significant. The bold values indicate p-values which are statistically significant.

**Table 4** shows logistic regression of adverse foeto- maternal complications and predictors of preeclampsia. In multivariate logistic regression model, having birth asphyxia [aOR = 27.14, 95% CI (1.80–409.83), *p* = 0.0170], previous caesarean section [aOR = 4.48, 95% CI (2.89–6.93), *p*<0.0001] and having foetal growth restriction (FGR) [aOR = 3.42, 95% CI (1.72–6.77), *p*<0.0001] were the independent foeto-maternal complication and predictors of preeclampsia **(Table 4).**

**Table 4:** Logistic regression model of adverse foeto-maternal complication and predictors of preeclampsia among study participants.

Variable	cOR (95% CI)	p-value	aOR (95% CI)	p-value
**Birth asphyxia**				
No	1.00		1.00	
Yes	10.56(1.47–75.80)	**0.0190**	27.14(1.80–409.83)	**0.0170**
**Prolonged labour**				
No	1.00		**-**	**-**
Yes	0.79(0.28–2.24)	0.6620	**-**	**-**
**PPH**				
No	1.00		**-**	**-**
Yes	1.00(0.45–2.23)	0.9980	**-**	**-**
**APH**				
No	1.00		**-**	**-**
Yes	5.30(0.96–29.25)	0.0560	**-**	**-**
**Mode of delivery**				
Previous SVD	1.00		1.00	
Previous C/S	4.60(3.00–7.05)	**<0.0001**	4.66(2.91–7.48)	**<0.0001**
**FGR**				
No	1.00		1.00	
Yes	3.17(1.75–5.75)	**<0.0001**	3.42(1.72–6.77)	**<0.0001**

PE, Preeclampsia; cOR, Crude Odd ratio; aOR, Adjusted Odd ratio; CI, Confidence interval; PPH, Postpartum Haemorrhage; APH, Antepartum Haemorrhage; SVD, Spontaneous Vaginal Delivery; C/S, Caesarean section; FGR, Foetal Growth Restriction; inf, infinity;1.00, reference. Binary logistic regression analysis performed to obtain odd ratios. *p*-value of < 0.05 was considered statistically significant.

**Table 5** depicts the combined independent risk predictors of preeclampsia. From the combination, Primigravida + FGR [aOR = 3.41, 95% CI (1.34–8.71), *p* = 0.0100], Primigravida + Previous C/S [a = 7.63, 95% CI (4.07–14.28), *p*<0.0001], and FGR+C/S [a = 21.21, 95% CI (9.14–49.23), *p*<0.0001] predicted an increased risk of preeclampsia compared to having only one of the predictors. Pregnant women being primigravida + FGR + Previous C/S) were 39-folds increased odds [aOR = 39.42, 95% CI (8.88–175.07, *p*<0.0001] of preeclampsia compared to having any of the two predictors [aOR = 6.612, 95% CI (3.62–12.08), *p*<0.0001] or having one predictor [aOR = 2.68, 95% CI (1.61–4.46), *p*<0.0001] **(Table 5).**

**Table 5:** Combined risk predictors of preeclampsia among study participants.

Variable	aOR (95% CI)	p-value
**Primigravida + FGR**		
0	1.00	
1	1.79(1.16–2.75)	**0.0080**
2	3.41(1.34–8.71)	**0.0100**
**Primigravida+ Previous C/S**		
0	1.00	
1	2.95(1.81–4.80)	**<0.0001**
2	7.63(4.07–14.28)	**<0.0001**
**FGR+C/S**		
0	1.00	
1	3.35(2.24–5.47)	**<0.0001**
2	21.21(9.14–49.23)	**<0.0001**
**Primigravida+ FGR + Previous C/S**		
0	1.00	
1	2.68(1.61–4.46)	**<0.0001**
2	6.61(3.62–12.08)	**<0.0001**
3	39.42(8.88–175.07)	**<0.0001**

aOR, Adjusted Odd Ratio; CI, Confidence interval; FGR, Foetal Growth Restriction; C/S, Caesarean section;1.00, reference. Binary logistic regression analysis performed to obtain odd ratios. p-value < 0.05 was considered statistically significant.

## Discussion

This study determined the prevalence and combined risk factors of preeclampsia among a cross-section of pregnant women attending antepartum care at a selected hospital and a health centre in the Mfantseman Municipality. Overall, the prevalence of preeclampsia in the present study was 8.8% prevalence which is consistent with 8.8% reported in a cross-sectional study conducted at Jos University Teaching Hospital, in Nigeria, [30]. Conversely, the prevalence reported in this study is higher than in other cross-sectional studies in Germany (2.31%) [31], Norway (3.0%) [32], Dilla, Ethiopia (2.23%) [33], and Accra, Ghana (7.03%, 7.9%) [8,34]. In contrast to other previous studies, the findings of this study found a lower prevalence as compared to 12.4% in Ethiopia [7], 16% in Nigeria [35], 25.4% in Ghana [36] and 51.9% in Ethiopia [37]. The differences in the prevalence could be attributed to different study settings, methodological variations, and seasonal variations.

Pre-eclampsia is typically regarded as a disease of the first pregnancy [38] which is caused by the immunological inefficiency between foeto-placental and maternal tissues found in the first pregnancy [39]. In this study, primigravida was independently associated with preeclampsia. Thus, primigravida women had 1.95 times increased risk of preeclampsia than multigravida pregnant women. On the contrary, primigravida was not associated with preeclampsia in a study by [40]. This variation may be attributed to the sociodemographic characteristics and the large sample size in this study.

Regarding maternal adverse complications, a cross-sectional study by [41] indicated that regardless of the preeclampsia status of the first pregnancy, previous caesarean section was significantly associated with increased risk of preeclampsia in the second pregnancy and this study agrees with our findings. In this study, having previous caesarean delivery was associated with 4.66 times increased risk of preeclampsia. The various uterine changes resulting from caesarean delivery may obstruct typical trophoblastic invasion and alter uteroplacental blood flow in succeeding pregnancies, resulting in preeclampsia in subsequent pregnancies [41]. This factor may contribute to the maternal adverse complications in preeclampsia as a result of previous caesarean sections in this study.

Another finding of this study was that foetal growth restriction (FGR) was associated with a higher risk of preeclampsia. This result is comparable to another cross-sectional study conducted by [42] that found a significant correlation between FGR and preeclampsia with an average birth weight of 2.64 kg for babies from PE mothers. In this current study, FGR was associated with 16.6% of low birth weight in women with preeclampsia.

Compared to normotensive mothers, neonates of preeclamptic mothers had considerably increased odds of asphyxia [aOR = 27.14, 95% CI = 1.80–409.83, *p* = 0.0170]. The result is consistent with others conducted in Gusau, Nigeria [43], India [44], Tigray -Ethiopia [45] and Bangkok Rajavithi hospital [46]. Other studies [47,48] found a stronger correlation between a poor obstetric history and asphyxia incidence compared to those with favourable obstetric history. Poor obstetric history may lead to foetal and maternal exhaustion which can increase the incidence of birth asphyxia.

Several studies have reported numerous risk factors of preeclampsia including maternal factors, environmental factors [7,49] etc. However, these studies have analysed these factors in isolation. In this study, the three identified risk factors for preeclampsia were combined to form four different sets. A multivariate analysis was performed to identify the predictive odds of the combined independent risk factors for preeclampsia. Results revealed the odds of Primigravida + FGR, Primigravida+ Previous C/S, FGR+C/S and Primigravida+ FGR + Previous C/S to be 3.41, 7.63, 21.21 and 39.42, respectively. Among these, the odds ratio of Primigravida+ FGR + Previous C/S was the highest and best, and further predicted that pregnant women co-habiting all the three independent factors were approximately 39 times increased risk of preeclampsia compared to a combination of any of these two factors. With this finding, pregnant women with these three risk factors are at a high chance or risk of adverse birth outcomes compared to having one or any of these two factors.

The strength of this study was that when these combined independent factors were used, the cumulative effect and predictive capacity and success of preeclampsia were improved. Although this study estimated the prevalence and identified some associated risk factors of preeclampsia, the study had some limitations. Firstly, the increase in preeclampsia prevalence may be as a result of the use of study participants from a hospital and healthcare centre. Secondly, the study was conducted in a divisional part of a region, thus the findings may not be nationally representative and could limit the generalizability of the findings to the broader population. Moreover, this study only showed associations between variables but could not establish causality. Therefore, it is not possible to determine whether preeclampsia is caused by any of the factors that were measured in the study, or whether those factors are merely correlated with the occurrence of preeclampsia. Furthermore, cross-sectional studies provide information about the prevalence of preeclampsia at a specific point in time, but they do not provide information about the timing of the occurrence of the condition or the course of the disease over time. Nevertheless, the findings of this study can facilitate larger, more comprehensive case-control or longitudinal studies in the country to gain better insights into the burden of preeclampsia in pregnancy.

## Conclusion

Among pregnant women in the Mankessim Municipality, the prevalence of preeclampsia is 8.8%. Factors such as primigravida, previous caesarean section (C/S) and foetal growth restriction (FGR) were independent risk factors of preeclampsia. Preeclampsia women are likely to have their babies associated with birth asphyxia. Pregnant women exhibiting multiple independent risk factors are the highest risk pregnant population likely to develop preeclampsia. It is incumbent on clinicians worldwide to monitor pregnant women with multiple risk factors and create targeted preventive measures including lifestyle modifications, such as diet and exercise, as well as pharmacological interventions, such as low-dose aspirin, calcium supplementation or antihypertensive medication; labetalol, nifedipine, or methyldopa should be implemented to manage and prevent the development of preeclampsia and other adverse pregnancy complications. These risk factors identified may also apply to pregnant women in other countries although the prevalence and impact of these factors may vary across different populations. Even though the physiology of pregnant women differs across population due to environmental differences, these differences do not underscore the aetiology of preeclampsia.

## Supporting information

S1 File (ODS)
